# A complete catalogue of human-infective RNA viruses

**DOI:** 10.1038/s41597-026-07281-5

**Published:** 2026-04-23

**Authors:** Feifei Zhang, Lu Lu, Liam Brierley, Helmi Hietanen, Mark E. J. Woolhouse

**Affiliations:** 1 Center for Digital Health and Artificial Intelligence, Peking University First Hospital, 8 Xishiku Street, Beijing, 100034 China; 2 Roslin Institute, Easter Bush, Midlothian, EH25 9RG UK; 3 MRC-University of Glasgow Centre for Virus Research, 464 Bearsden Road, Glasgow, G61 1QH UK; 4 Institute of Immunology & Infection Research, University of Edinburgh, Ashworth Laboratories, Charlotte Auerbach Road, Edinburgh, EH9 3FL UK; 5 Usher Institute, University of Edinburgh, Ashworth Laboratories, Charlotte Auerbach Road, Edinburgh, EH9 3FL UK

**Keywords:** Viral infection, Biological sciences

## Abstract

RNA viruses are a pervasive and serious threat to human health. Here, we provide an updated catalogue of all 239 human-infective RNA virus species recognised by the end of 2024. Based on extensive literature searches, we provide metadata on the date and location of the first reported case of human infection, transmissibility in human populations, transmission route(s) and host range. We also provide links to publicly available genome sequence data. The dataset can be used in studies of the relationships between virus traits and public health threat, the phylogenetics of those traits, the geography of emerging RNA viruses, projections of future discovery rates, and estimates of viral diversity.

## Background & Summary

Infectious diseases caused by RNA viruses – such as influenza, measles and AIDS – are responsible for a huge global health burden^1^. RNA viruses are also prominent among emerging pathogens with epidemic or pandemic potential: SARS-CoV-2 and Oropouche virus being recent, high-profile examples.

Our research group first published a catalogue of human-infective RNA viruses (and other infectious agents) in 2001^2^. However, the list of viruses is continually changing: new species are discovered to infect humans almost every year and viral taxonomy is repeatedly revised. At the same time, knowledge of these viruses grows, more virus genomes are sequenced, and new information emerges on key traits such as transmissibility in humans, transmission routes and non-human hosts that act as reservoirs. For these reasons, we have regularly refined our methodology and updated the catalogue, most recently in 2018^3^.

Here, we are publishing a further updated and extended version of the 2018 dataset. The new version^4^ takes into account revisions to RNA virus taxonomy, includes more than 20 new species added in the intervening years, updates information on virus traits, provides new information on the location of first discovery, and provides links to virus genome sequence data (where available). A fuller comparison of the data fields making up the two datasets is given below. We stress that the dataset will continue to evolve as new information is published – this version represents a snapshot of the state of knowledge as of December 2024, compiled using the methodology described below.

The new dataset^4^ lists 239 ICTV-recognised, human-infective RNA virus species, an increase of 25 on the corresponding 2018 count. This reflects the continuing discovery of RNA viruses (13 entirely novel species, 6 from China) and changes to species classifications over recent years, plus modifications to our inclusion criteria (see below).

**Fig. 1:**
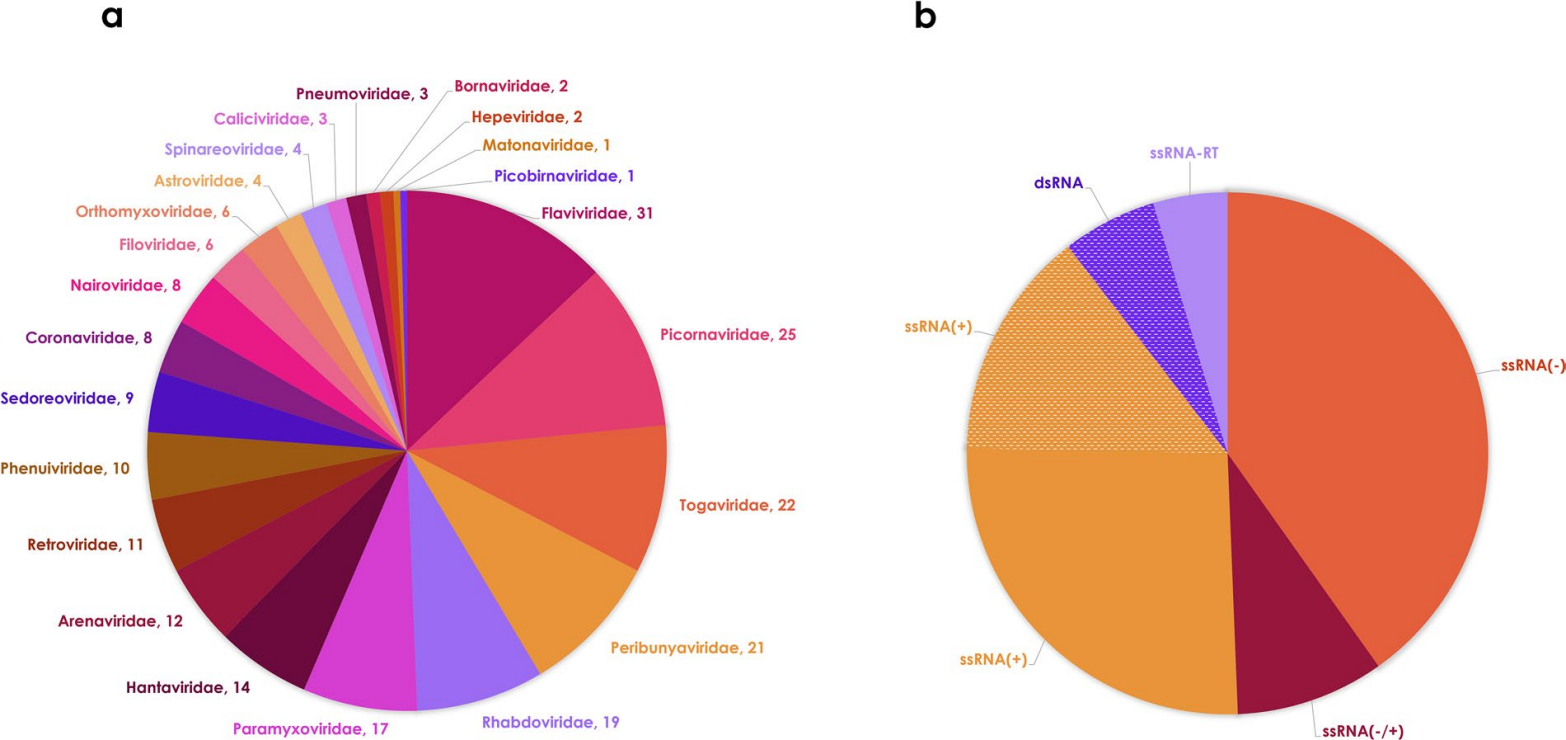
Taxonomy of human-infective RNA viruses. (**a**) Pie chart showing the distribution of 239 species across 23 families. (**b**) Pie chart showing the distribution of 239 species across five genome types and whether enveloped (solid) or not enveloped (shaded), noting that some ssRNA( + ) viruses are enveloped and some are not.

The 239 species are currently classified in 61 genera from 23 families (Fig. 1a), an increase of six genera and two families. Four of the genera (Rocahepevirus, Deltacoronavirus, Norwavirus and Parahenipavirus) are represented only by species discovered since 2018. The other added genera, and both the added families, result from taxonomic revisions by ICTV applied to viruses known prior to 2018.

Five genome types are represented in the dataset, with 80% of species being enveloped (Fig. 1b). Whole genome sequences are currently available for 207 species.

**Fig. 2:**
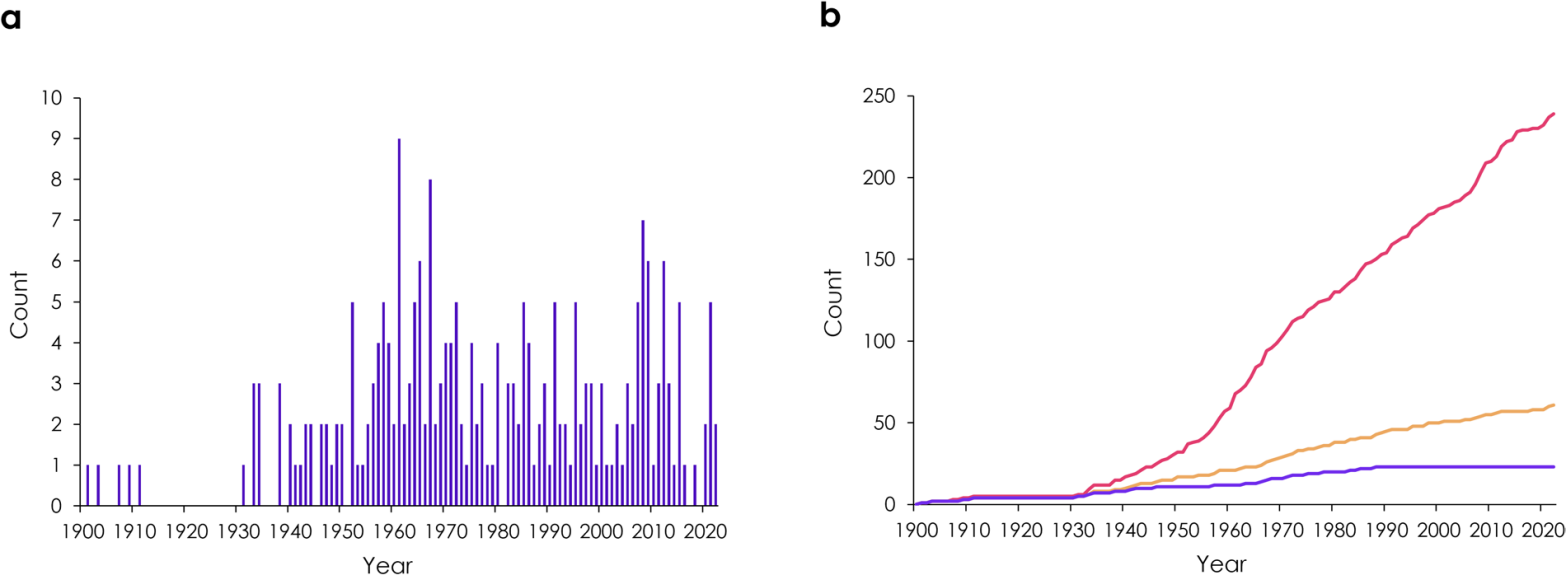
Accumulation over time of human-infective RNA viruses. (**a**) Time line of annual counts of new species. (**b**) Cumulative counts of species (red line) and of genera (gold) and families (purple) containing RNA virus species known to infect humans.

The annual count of new and (currently) ICTV-recognised, human-infective RNA virus species is shown in Fig. 2a. Figure 2b depicts the accumulation of species over time, as well as the accumulation of genera and of families containing one or more human-infective RNA virus species. The first human RNA virus – Yellow fever virus – was reported in 1901. The number of species increases slowly up to the mid-1950s and somewhat faster thereafter. By the end of the 20th century 178 species had been identified, and in the 21st century so far, a further 61 have been added. By decade, the 1960s contributed markedly the greatest number of new species (42). Next was the 2000s (31) but the rate fell again in the 2010s.

**Fig. 3:**
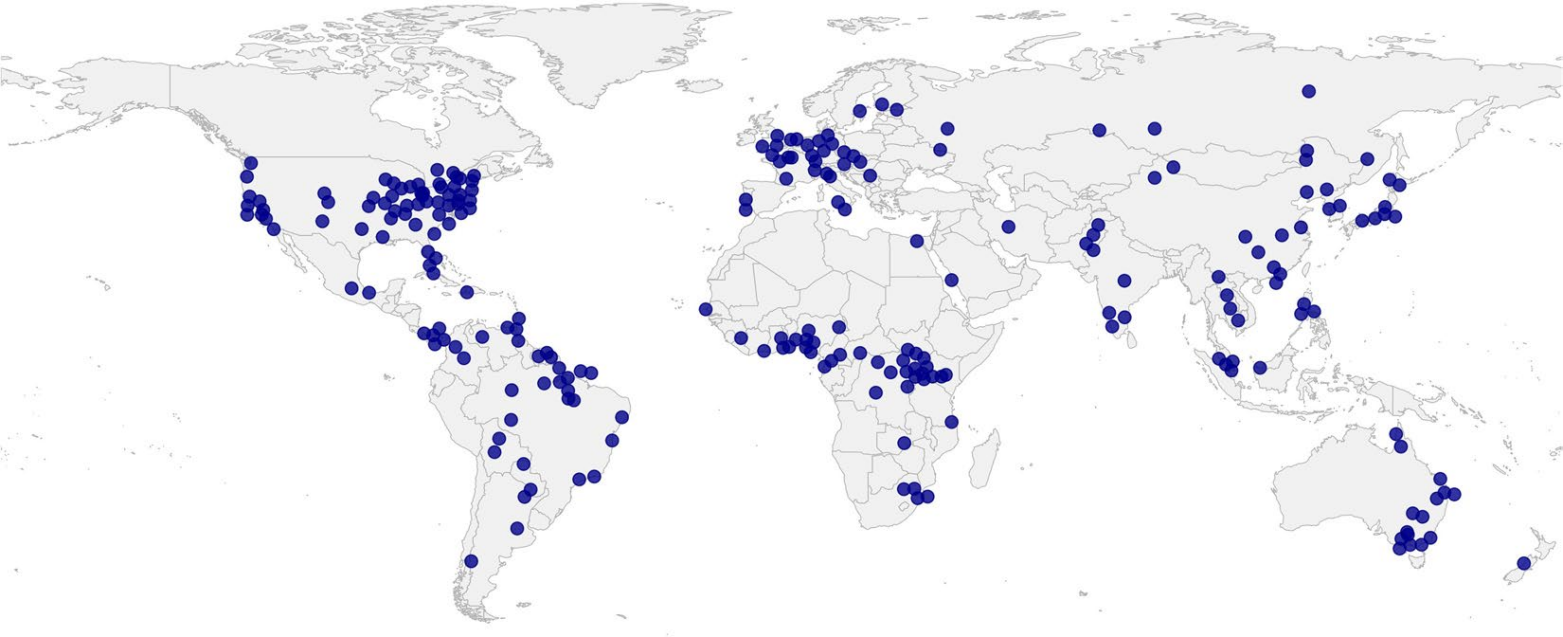
Map of locations of first reported human cases of all 239 currently recognised human-infective RNA virus species. Locations are positioned by latitude and longitude, but jittered where necessary to avoid visual overlap. Exact geolocations for all first reported cases are provided in ref. ^4^ (columns L and M).

New human viruses have been reported on multiple occasions from every world region except Antarctica. Ninety-nine new species were first reported from the Americas, 81 from Eurasia, 43 from Africa and the Middle East and 16 from Oceania. A map of discovery locations is provided as Fig. 3.

**Fig. 4:**
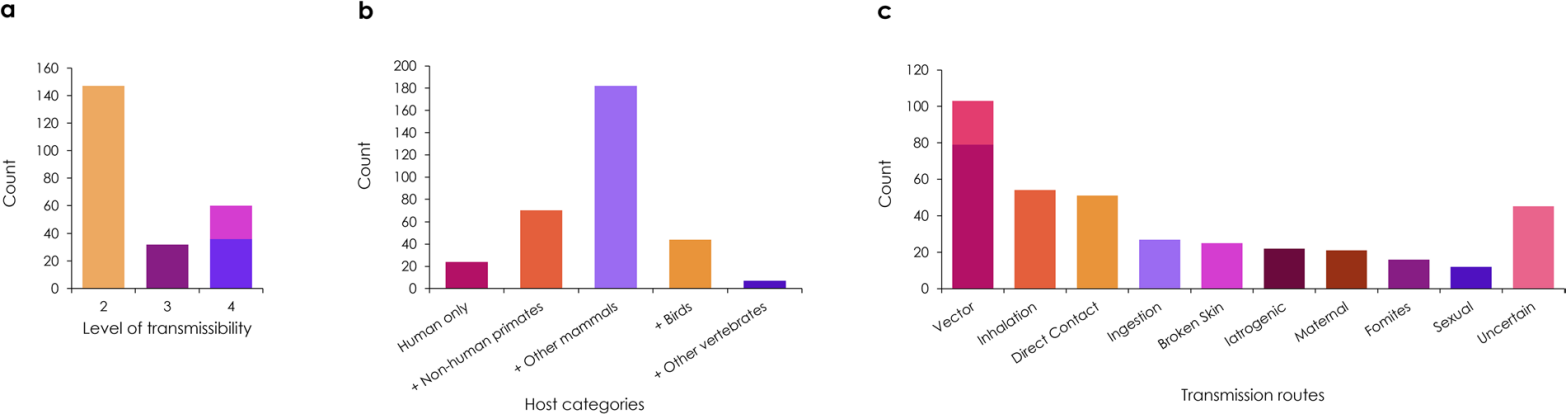
Ecological characteristics of 239 human-infective RNA virus species. (**a**) Species counts by transmission level in humans: 2 (strictly zoonotic); 3 (self-limiting outbreaks); 4 (epidemic or endemic in humans). Level 4 is divided into species that are naturally infect non-human hosts (4a) (including one species suspected to infected non-human hosts – see main text) and those that have only been reported to infect humans (4b). (**b**) Species count by host category, distinguishing species only known to infect humans from those that also naturally infect non-human primates, other mammals, birds or other vertebrates. Some species naturally infect more than one non-human host category. Suspected host categories are not included. (**c**) Species count by known transmission routes, noting that some species can be transmitted by more than one route. Vector-borne category distinguishes dipteran vectors (dark purple) and tick vectors (light purple). Suspected routes are not included, so for some species the transmission route to or between humans remains uncertain.

Human-infective RNA viruses are variably transmissible (by any natural route) from person to person. At one end of the scale are 147 strictly zoonotic species (62% of the total), where all human cases are believed to have been acquired through transmission from a non-human host – we refer to these as Level 2 viruses. At the other end of the scale are 60 species that are endemic in humans or are capable of epidemic spread in humans – we refer to these as Level 4. Thirty-six Level 4 species have known, natural, non-human hosts, so are considered zoonoses or anthropozoonoses – we refer to these as Level 4a. Twenty-four Level 4 species have no known, natural, non-human hosts – we refer to these as Level 4b. In between is a category of 32 viruses that have been reported to transmit within human populations but, to date, have been confined to self-limiting outbreaks – we refer to these as Level 3. Numbers of Level 2, 3, 4a and 4b RNA virus species are shown in Fig. 4a.

Data on RNA virus host range are patchy and incomplete. Here, we distinguish five broad categories: natural infections known only from humans; naturally infects non-human primates as well as humans; naturally infects non-primate mammals as well as humans; naturally infects birds as well as humans; naturally infects other vertebrates (mainly reptiles) as well as humans. Forty-four species (18%) naturally infect birds; only 7 species (3%) infect other vertebrates. There are 24 virus species (10%) that are currently only known to infect humans. Numbers of virus species in these categories are shown in Fig. 4b, noting that some species can infect more than one category of non-human host.

RNA viruses can be transmitted to humans in a variety of ways. We distinguish nine different transmission routes; the frequency of each is shown in Fig. 4c, noting that the majority of viruses are known to be transmitted by more than one route. A total of 103 (43%) human RNA virus species are known to be transmitted by vectors, more than by any other route. Natural vectors are invariably either dipterans (biting flies such as mosquitoes, midges and sandflies) or ixodids (ticks) but not both. For a minority of vector-borne viruses (18 species), transmission has also been reported by one or more other routes. By our criteria, 45 virus species have uncertain transmission route(s) to or between humans – these are mostly rare, strictly zoonotic viruses.

Previous versions of the dataset have been used in spatial and temporal mapping of virus discovery^5–7^, to provide phenotype data in phylogenetic analyses^8^, and in comparative studies of viral pathogenicity^9^. In the future, we hope the dataset will be valuable for research on the diversity of human-infective RNA viruses, comparative studies of virus traits, and of the identification of virus traits associated with risk of becoming an emerging public health threat. Updated counts of species, genera and families could be used to update projections of the discovery curve using statistical modelling^5^ or biodiversity estimators^10^. An important first step would be to establish whether or not the rate of discovery is rising (as a potential consequence of increased effort and technologies such as metagenomics) or falling (which would be consistent with suggestions that the pool of human RNA viruses is smaller than has commonly been assumed^5,10^). Figure 2 appears more consistent with the latter, but formal analysis is required.

Suggestions to the authors for updating and adding to the information contained in the dataset are welcome. We accept additions, corrections or amendments if they can be linked to a publication referenced in scientific literature databases, but not if they are based on unpublished observations.

## Methods

The methods used to create the dataset are largely as described in our 2018 publication^3^. Here, we briefly reiterate key elements and detail modifications to the earlier methodology. No new code was used in compiling this or any other version of the dataset.

Literature searches were performed every 1–3 years between January 1999 and December 2024. Any inconsistencies or ambiguities in the interpretation of the literature were resolved by reaching consensus following independent assessments by at least two individuals (always including one or more of the authors). Throughout, a positive data entry implies positive evidence from the scientific literature. A negative data entry may reflect the absence of evidence rather than evidence of absence, and negative data entries may be revised as further studies are published.

Where there is no direct evidence for traits of individual virus species, it is sometimes possible to infer those traits from closely related species. Where virus traits are inferred rather than directly observed this is indicated in the dataset.

We note that there may be variability in traits of interest within as well as between virus species. For example, there is huge variation in the epidemiology of subtypes of Influenza A virus: subtypes such as H1N1 and H3N2 spread easily within human populations, whereas avian influenza viruses such as H5N1 and H7N9 are almost exclusively zoonotic, and other subtypes do not infect humans at all. Although Influenza A virus is by far the most variable in this regard, other virus species have distinct subtypes that differ in traits such as antigenicity (e.g. Dengue virus) or host preference (e.g. Rabies virus). Here, we combine the traits of all identified subtypes to make up the traits of that virus species as a single taxonomic unit.

We provide sources for the data in the dataset. These are PMID numbers where available, otherwise ISBN numbers or direct links to cited web sites.

### Identification of human-infective species

The criterion for inclusion in the dataset is a peer-reviewed primary case report providing robust evidence of natural human infection with an RNA virus species recognised by the International Committee for the Taxonomy of Viruses (ICTV)^11^. Unintentional iatrogenic infections or infections acquired through occupational laboratory exposure were considered ‘natural’ human infections, but intentional infections (e.g. experimental inoculation) and *in vitro* infections (e.g. cultures in human cells) were excluded.

Literature searches covered multiple reference databases (Web of Science, Google Scholar, PubMed and Scopus), supplemented by secondary sources such as the WHO and US CDC web sites, moderated web resources such as ProMed, one comprehensive review article^12^ and the Human Virus Database^13^. We also followed up primary references linked to RNA virus genome sequence data in NCBI (www.ncbi.nlm.nih.gov/nucleotide/), where this suggested human infection. Structured searches typically included the following key words: [virus name] and human and (case* or patient* or infection* or disease* or outbreak* or zoono*). Searches for newly reported viruses added the key word term: (new or novel).

Where a virus species has one or more common names in addition to its scientific name, and/or when different scientific names have been used previously (information available from ICTV^11^), these alternative names were all included in literature searches. Only evidence of infections with the named virus species, or strains/variants falling within that species, was accepted; reports of “[virus name]-like” or “potential [virus name]” infections were excluded.

All viruses in the dataset were classified as a species by the ICTV as of December 31st 2024. The main ICTV criteria for defining RNA virus species are: relatedness based on sequence data; serological cross-reactivity; host range; and transmission route^11^. We note that ICTV classifications to species, genus and family have been revised frequently since this study began, and we anticipate this will continue.

In a departure from our previous catalogues, we now specifically exclude viruses of the genus Deltavirus. These are small, circular RNA viruses that are atypical in requiring helper viruses (e.g. hepatitis B) to infect human cells and are described by ICTV as “viroid-like”^11^. Their biology is therefore not directly comparable with the true viruses in the dataset.

The final decision to accept a virus species as human-infective was based on our own expert review. That review took the following into consideration: virus identifiable as a currently recognised ICTV species or subtype of a recognised species; diagnostic method(s) used to identify human infection; number of reported infections; number of publications independently reporting infection; expressed author confidence in identity and infectivity of virus. Pathogenicity was not considered a criterion for human infectivity. This exercise is often challenging and we maintain a list of possible human viruses that are not currently included in the dataset but have been suggested as infecting humans; the list is periodically revisited to check for relevant new evidence in the literature.

Viruses identified using serology are particularly problematic. Serological evidence would not now be considered sufficient to declare a new human virus, but was used extensively in the 20th century. We have attempted to minimise false positives from serological reactions with related viruses by only accepting evidence from studies where cross-reactivity was explicitly ruled out. This still leaves > 30 species in the dataset that were first identified using serology. Eight of these species have: i) only been detected in humans on the basis of serological evidence; ii) only been reported in humans in a single, published study; iii) not been detected in humans since the year 2000. For five of these viruses the evidence of human infection is particularly weak: Mobala virus (a Mammarenavirus reported from the Central African Republic in 1985); Gadgets Gully virus (a Flavivirus reported from Australia in 1991); Patois virus (a Peribunyavirus reported from Mexico in 1972); Maraba virus (a Rhabdovirus reported from Brazil in 1984); and Whataroa virus (a Togavirus reported from New Zealand in 1964). Nonetheless, all of these viruses are reported as human-infective in the peer-reviewed literature, and all come from genera with multiple human-infective species, so we have retained them in the dataset, noting that this small number of diverse viruses does not unduly influence the summary statistics presented here.

In addition to the common name of the virus, we include the current ICTV species, genus and family names, together with a link to ICTV taxonomic history of the species. We also include information on whether or not the virus is enveloped and its genome type (double stranded, negative single-stranded, ambisense single-stranded, positive single-stranded or single-stranded reverse transcriptase). The distributions of species count by family and by envelope/genome type are shown in Fig. 1.

For each virus species, we provide a link to a whole genome sequence in the NCBI database^14^. Where possible, the sequence referenced is the ICTV ‘exemplar sequence’ for that virus species. Where no link is provided no full genome sequence is available (though partial sequences are available in some instances).

### Discovery date and location

Each virus species is linked to the first published report of human infection in the scientific or medical literature. We used structured searches (as described above) to confirm the absence of earlier reports. The linked reference provides available details of detection and identification methods used. We took the date of discovery of human infection as the date of publication of the first reported case rather than the date of sampling (which is often unavailable).

Based on these discovery dates, we plot the annual count of new and (currently) ICTV-recognised, human-infective RNA virus species up to 2024 in Fig. 2a. In Fig. 2b, we show the accumulation of species over time, and repeat this exercise for the accumulation of genera and of families containing human-infective species.

We note that there is often a lag of several years between a novel virus being reported to infect humans and that virus being recognised as a new species, or not, by ICTV. For this reason, the data in Fig. 2 may change in the future, particularly for the most recent years.

We have added information on the locations of virus discoveries – this was not included in the 2018 version of the dataset. Location information is provided as the name of the country where the first human case was found, plus latitude and longitude of the discovery site. Following a previously published methodology^6^, latitude and longitude are given as the central point of a county, region, district or country where that is most precise discovery site information available for some viruses. Latitude and longitude are used to map virus discovery locations (Fig. 3). This updates a previously published map^6^.

### Transmissibility in humans

Data on transmissibility in humans have been updated from a previous study^15^; full details of the search strategy are provided in the Technical Appendix to that publication.

Each virus is classified according to its known level of transmissibility in human populations. Transmission may be via a natural route, including by arthropod vectors, or as unintentional iatrogenic transmission, but deliberate laboratory exposures are excluded. In keeping with previous usage^15^, transmissibility is assigned to one of four levels. Level 2 indicates human infective but not transmissible between humans. Level 3 indicates transmissible from one human to another (by any natural route including arthropod vectors), but so far restricted to self-limiting outbreaks. Level 4 indicates viruses that are endemic in human populations or have caused epidemics or localised outbreaks numbering 1000 s of cases. Where virus species include subtypes known to have different levels of transmissibility, e.g. Influenza A virus, the highest level is assigned.

The distribution of species by these different levels of transmissibility is given in Fig. 4a. The majority (62%) of human-infective RNA viruses are Level 2, strictly zoonotic. We note that Level 4 includes viruses that are known to naturally infect non-human hosts (Level 4a) and viruses that are only known to infect humans (Level 4b). O’nyong-nyong virus has caused sporadic large outbreaks in humans and is believed to have an as yet unidentified non-human (probably primate) reservoir, so is designated 4a*.

### Host range

Literature searches for information on host range were conducted using Web of Science, Google Scholar, and Scopus. Structured search key words were: ([virus name] and all synonyms listed by ICTV separated by ‘or’) and (host or range or reservoir or animal or mammal or bird or avian or reptile* or amphibia* or vertebrate). Data on host range was obtained from peer-reviewed primary publications where available, supplemented by information from the Global Mammal Parasite Database^16^ and two publications reporting previous surveys of host range^17,18^.

For each virus, we record the known range of non-human hosts, categorised as follows: non-human primates; other mammals; birds; other vertebrates. The distribution of human RNA virus species by host range is shown in Fig. 4b. Nine species (seven Level 2, one Level 3 and one Level 4) are suspected to have a non-human reservoir, but none has yet been identified. These viruses are recorded as not human only; suspected non-human host categories are indicated by 1*. This subset includes all four human tibroviruses, where related viruses have been found in chimpanzees and cattle, so these species are listed as potentially having non-human primate and other mammal hosts (indicated as 1*).

### Transmission route

Literature reviews for transmission route data were conducted within Web of Science, Google Scholar and Scopus. Structured search key words were: ([virus name] and all synonyms listed by ICTV separated by ‘or’) and (transm* or *borne or vector).

We record all reported routes of transmission for each virus species, distinguishing vector-borne, inhalation, ingestion, sexual contact, iatrogenic (including blood transfusion), fomites, broken skin (including wounds and bites), maternal (mother to offspring transmission in utero or via breast milk) and direct contact (any form of close physical contact), regardless of relative frequency. We present these data first for vector-borne transmission and the type of vector (dipteran or ixodid), then for the other 8 possible transmission routes.

Where there is positive evidence of a transmission route to or between humans this is indicated by 1. If there is positive evidence for any transmission route to or between humans then all other routes are designated 0, even when they may be suspected/potential. Parainfluenza virus 5 is designated 0* as it has been found in ticks but no other congeneric or confamilial species are transmitted by this route. However, if there is no good evidence for any specified transmission route then all suspected/potential routes are designated 1*. 1* entries are obtained either from suggestions made in the scientific literature or are inferred from congeneric viruses. Only entries of 1 (and not 1*) are included in the count of transmission routes. This leaves 45 virus species with uncertain transmission route(s) to or between humans. Figure 4c shows the numbers of virus species transmitted by each route.

### Comparison of datasets

The new dataset describes 26 additional human-infective virus species but excludes the single Deltavirus present in the 2018 version. The substantive new data fields are: i) links to genome sequence data (where available) and ii) information of the geolocation of the discovery site. These allow the data to be more easily integrated with, respectively, phylogenetic studies and mapping studies. We also provide additional information on nomenclature and (where relevant) vector type.

All new data fields are identified in the Data Records section below. We have discarded fields in the 2018 version referring to discovery by serology, and redundant fields relating to person-to-person transmission and host breadth. In addition, we have amalgamated fields relating to non-mammal/non-bird hosts as ‘Other vertebrates’.

Since 2018, there have also been a small number of updates to the data available on transmission level, host range and transmission route. We have also updated the links to publications databases. Genus and family names reflect changes to ICTV classification between 2018 and 2024.

## Data Records

The dataset is available in xlsx format via figshare^4^. It contains information for all 239 currently ICTV-recognised species of RNA virus for which there is published evidence of human infectivity in the following fields.

Column A (new): Common name. Human-infective RNA viruses identified as described in Methods.

Column B: Current ICTV species name. From the ICTV web site (https://talk.ictvonline.org/).

Column C: Current ICTV genus name. From the ICTV web site (https://talk.ictvonline.org/).

Column D: Current ICTV family name. From the ICTV web site (https://talk.ictvonline.org/).

Column E: ICTV taxonomic history. As ICTV taxon ID number and hyperlink to URL.

Column F: Enveloped. Whether or not the virus is enveloped as 1 or 0. From the ICTV web site (https://talk.ictvonline.org/).

Column G: Genome type. Five types: dsRNA = double stranded RNA; ssRNA(-) = negative single-stranded RNA; ssRNA(-/ + ) = ambisense single-stranded RNA; ssRNA( + ) = positive single-stranded RNA; ssRNA-RT = single-stranded reverse transcriptase RNA. From the ICTV web site (https://talk.ictvonline.org/).

Column H (new): Sequence ID. As NCBI GenBank accession number. Sequence ID is only provided if a whole genome sequence is available, though for segmented viruses the ID number may link to a single genome segment. Partial sequences are available for some of the remaining viruses, but those are not included here.

Column I: Discovery date. As calendar year of first published report of virus infecting humans. Obtained from literature review as described in Methods. Range from 1901 to 2022.

Column J (new): Discovery country. Country where virus was first discovered. Obtained from literature review as described in Methods.

Column K (new): Discovery region. As North America, Caribbean, South America, Sub-Saharan Africa, Oceania, Asia, Europe or North Africa/Middle East.

Columns L and M (new): Latitude and longitude (of discovery location). Reported location may be a town/village, county/region/district or country. Where necessary, latitude and longitude are given as a near-central point of the designated area.

Column N: Discovery reference. PubMed ID where available and hyperlink or citation to reference.

Column O: Transmission level. As 2, 3, 4a or 4b where: 2 = human infective but not transmissible between humans; 3 = transmissible from one human to another by any natural route including arthropod vectors, but restricted to self-limiting outbreaks; 4a = endemic in human populations or has the potential to cause major epidemics, and is known to naturally infect non-human hosts; 4b = endemic in human populations or has the potential to cause major epidemics, and is only known to infect humans [ignoring arthropod vectors]. Asterisk indicates inferred from closely related species. Obtained from literature review as described in Methods.

Columns P to R: Transmission level reference(s). PubMed ID where available and hyperlink to reference.

Column S: Vector. Whether or not the virus is transmitted to and/or between humans by an arthropod vector. As 1, 1*, 0 or 0*. 1* and 0* indicate inferred from closely related species or presumed to be the same as the animal-to-animal route.

Column T (new): Vector order. Whether the main vector is a Dipteran (mosquito, midge or sandfly) or Ixodid (tick). Asterisk indicates inferred from closely related species.

Columns U and V: Vector reference(s). PubMed ID where available and hyperlink to reference.

Columns W to AD: Transmission routes. Whether or not transmission to and/or between humans is known via any route other than an arthropod vector (see Columns S and T). Categories are: inhalation; ingestion; sexual contact; iatrogenic; fomites; broken skin; maternal; direct contact. All as 1, 1* or 0 where 1* indicates inferred from closely related species or assumed to be the same as the animal-to-animal route (used only for species with no confirmed transmission route). Obtained from literature review as described in Methods.

Columns AE and AF: Transmission route reference(s). PubMed ID where available and hyperlink to reference.

Columns AG to AK: Host range. Whether or not the virus is known to be capable of infecting each of five host categories: only humans; non-human primates; other mammals; birds; other vertebrates. All as 1, 1* or 0 where asterisk indicates inferred from closely related species. Obtained from literature review as described in Methods.

Columns AL and AM: Host range reference(s). PubMed ID where available and hyperlink to reference.

## Technical Validation

Information on discovery date and location, transmission level, host range and transmission route are all supported by a reference from the scientific literature. Links to the supporting references are provided as the PubMed ID if available or as a direct link (hyperlinks included in the dataset columns N, P, Q, R, U, V, AE, AF, AL, AM).

## Data Availability

The dataset is available in xlsx format via figshare (10.6084/m9.figshare.30094726)^4^. It contains information for all 239 currently ICTV-recognised species of RNA virus for which there is published evidence of human infectivity. There are no restrictions on the use of dataset as it contains no personal information and uses only previously published sources. The dataset is licenced as Creative Commons Attribution 4.0 International.

## Code availability

No custom code was used in this study.
